# Nanoparticles Containing Tamarind Isolate Protein Potentiate the Satiety without Promoting the Anti-Inflammatory Effect in a Preclinical Model of Diet-Induced Obesity

**DOI:** 10.3390/foods11213526

**Published:** 2022-11-05

**Authors:** Rafael O. A. Costa, Isaiane Medeiros, Jaluza L. C. De Queiroz, Lídia L. R. Matias, Mayara S. R. Lima, Gerciane S. De Oliveira, Ana Júlia F. C. Aguiar, Izael S. Costa, Eloyse Mikaelly de S. Silva, Nicolle Caroline S. Dos Santos, Thaís S. Passos, Ana Heloneida De A. Morais

**Affiliations:** 1 Biochemistry and Molecular Biology Postgraduate Program, Biosciences Center, Federal University of Rio Grande do Norte, Natal 59078-970, RN, Brazil; 2 Nutrition Postgraduate Program, Center for Health Sciences, Federal University of Rio Grande do Norte, Natal 59078-970, RN, Brazil; 3 Nutrition Course, Center for Health Sciences, Federal University of Rio Grande do Norte, Natal 59078-970, RN, Brazil; 4 Department of Nutrition, Federal University of Rio Grande do Norte, Natal, 59078-970, RN, Brazil

**Keywords:** trypsin inhibitor, tamarind, encapsulation, antitrypsin activity, pH conditions, thermal treatment

## Abstract

The study aimed to evaluate the nanoparticles (ECW) containing tamarind trypsin inhibitor (TTI) concerning the storage effect under different conditions on antitrypsin activity and the bioactive potential in a preclinical model. ECW was exposed to different pH and temperatures to evaluate the interaction between TTI and its encapsulating agents, monitored by antitrypsin activity. Wistar rats (*n* = 25) with obesity induced by diet were divided into groups: untreated; treatment with nutritionally adequate diet; treatment with nutritionally adequate diet and ECW/12.5 mg/kg; treatment with ECW/12.5 mg/kg; and treatment with TTI/25 mg/kg. The groups were evaluated over ten days with regards to satiety, zoometric, biochemical, and inflammatory parameters, using ten times less TTI (2.5 mg/kg) contained in ECW. TTI was protected and encapsulated in ECW without showing residual inhibitory activity. Only at gastric pH did ECW show antitrypsin activity. At different temperatures, it showed high antitrypsin activity, similar to TTI. The animals treated with ECW had significantly reduced body weight variation (*p* < 0.05), and only TTI treatment reduced the inflammatory parameters significantly (*p* < 0.05). The study showed that by using lower concentrations of TTI in ECW it was possible to perceive promising effects with perspectives of use in functional products for managing obesity and its complications.

## 1. Introduction

In recent decades, the world population has increased its consumption of foods with high caloric density, low satiety power, and easy digestion and absorption. These provide an excessive intake of low-quality nutrients, which are associated with genetic and environmental factors, and cause metabolic changes, such as obesity and related complications [1].

Therefore, searching for new strategies for managing obesity is the reason for numerous studies. An alternative for the treatment of obesity is the use of peptidase inhibitors combined with diet [2]. Thus, tamarind seeds constitute an interesting source of trypsin inhibitors [3,4]. Because of its potential, in several studies by the research group on *Nutrição e Substâncias Bioativas Aplicadas à saúde* (NutriSBioativoS) at UFRN, in Brazil, the effects of a trypsin inhibitor isolated from tamarind seeds, called TTI, have been evaluated in eutrophic and obese animals. Its role in satiety, improving metabolic parameters, and reducing inflammation has been demonstrated [5,6,7,8,9].

The targeted delivery of peptides and proteins is of great interest to the food and pharmaceutical industry since the bioactive properties of these molecules can be impacted due to different exposure conditions of administration. These conditions can be influenced by pH, temperature, and ionic strength, and denaturing conditions, enzymatic action, and mechanical action acting directly on its structure, affecting the activity [10]. Notably, some proteins are susceptible to degradation in a highly acidic environment rich in proteases, such as in the human stomach [11].

Encapsulating these actives of a protein nature can guarantee better stability for these molecules, protecting them against the proteolytic environment of the gastrointestinal tract. Proteins with bioactive functions may lose activity due to changes in the three-dimensional structure and hydrolysis in undesired situations, compromising biological function and efficacy [12].

Queiroz et al. [13] performed a comparative study of TTI nanoencapsulation using three different encapsulating agents (chitosan—ECH), isolated milk protein—EWPI, and the combination of these two materials—ECW). The nanoparticles with greater chemical stability and potentiated antitrypsin activity were synthesized by combining encapsulating agents (ECW), which showed a strong chemical interaction between the inhibitor and encapsulating agents. Thus, ECW proved to be an excellent vehicle to potentiate TTI functionalities, for example, satietogenic, anti-inflammatory, or other activities related to obesity treatment.

These nanoparticles were showed to be promising for technological and clinical application since they presented stability in different pH and temperature conditions [13]. On the other hand, the effect promoted by conditions related to physiological and storage temperature and physiological pH (oral, gastric, and intestinal), without the influence of enzymatic action, on the antitrypsin activity of TTI contained in ECW still needs to be investigated.

According to Costa et al. [14], there have been no reports of in vitro and in vivo toxicity attributed to ECW under the conditions studied. These preliminary results were essential to ensure the use of this nanoformulation in preclinical studies and to investigate the bioactive effects. Thus, studies were conducted with ECW administered in an experimental model of obesity induced by diet using Wistar rats. It improved hepatic parameters and carbohydrate metabolism, reducing glycemia [15,16].

However, in these studies, the ECW potential on the effect on satiety, inflammatory parameters, and at the hormonal level, such as leptin concentration, was not evaluated. It is noteworthy that the impact of TTI on satiety did not lead to weight changes when administered to obese animals [6]. As inflammation is a characteristic condition of obesity, investigating the behavior of inflammation markers with ECW administration, in addition to its action on leptin, is of great importance as it provides data to strengthen the possibility of using this molecule as an alternative in the treatment of obesity.

In this perspective, understanding the function of encapsulated peptides and proteins is essential. Since the chemical interactions between the system components may change, new functionalities, which were not observed in the molecule before encapsulation, may become evident. Therefore, evaluating the effect of different temperature, pH, and time conditions in TTI contained in ECW, and its functionality in in vivo models, is of interest. There is still much to study, especially to understand its effectiveness in reducing the metabolic conditions associated with obesity. This study will provide a better understanding of the applicability perspective of these nanoparticles containing TTI, making it possible to consider their future use in food and pharmaceutical products.

From the bioactivities of TTI, it is expected that the same nanoencapsulated ECW administered to Wistar rats with diet-induced obesity may promote effects on satiety and metabolic and inflammatory parameters related to this condition.

## 2. Materials and Methods

### 2.1. Isolation of the Trypsin Inhibitor from Tamarind Seed (TTI)

Tamarind (*Tamarindus indica* L.) was obtained and botanically identified by the Brazilian Institute for the Environment and Renewable Natural Resources (Ibama) in a seed bank located in Natal/RN (Brazil) and registered in the Management of the National Genetic Heritage and Associated Traditional Knowledge (SisGen) under the number AF6CE9C.

All tests to obtain the TTI were performed according to the standardized methodology and adapted by Carvalho et al. [6]. Isolation monitoring was monitored through inhibitory activity against trypsin [17], using 1.25 mM BApNA (Nbenzoyl-DL-arginine-p-nitroanilide) as substrate. Antitrypsin activity was expressed as a percentage (%), and specific activity was determined. The inhibition unit (IU) was established by the difference between the total enzymatic activity of trypsin and the enzyme activity with EB, F2, and TTI at absorbances from 0.01 to 410 nm (DR 5000™ UV-Vis Spectrophotometer—Hach). Thus, 1 IU was defined as the amount of inhibitor that decreases the absorbance by 0.01 optical density under the trypsin assay conditions.

The denaturing polyacrylamide electrophoresis gel (SDS-PAGE) at 12% was used to evaluate the isolation of the TTI and estimate its molecular mass, as described by Laemmli [18]. The molecular mass marker ECLTM Rainbow™ Marker—Full Ranger (Amersham™) (12,000–225,000 Da) was used. After electrophoresis, the proteins were revealed in a dye solution composed of 10 mL of methanol and 40 mL of Coomassie blue R-250.

### 2.2. Synthesis and Physicochemical Characterization of Nanoencapsulated Trypsin Inhibitor from Tamarind Seeds (ECW)

This procedure followed the method established by Queiroz et al. (2018). The formulation was synthesized following the ratio, TTI–purified chitosan–whey protein isolate of 1:2:2 *w*/*w*/*w*. The whey protein isolate was supplied by Alibra Ingredients Ltd.a^®^ (Parque Industrial—M.C. Rondom/PR/Brazil), and the chitosan was purchased from Sigma-Aldrich^®^ (St. Louis, MO, USA).

The encapsulated powder was characterized by Scanning Electron Microscopy (SEM) (Zeiss Auriga with FEG type electron source), Fourier Transform Infrared Spectroscopy (FTIR) (Shimadzu, FTIR-8400S model, IRAFFINITY-1, and IRSOLUTION software, version 1.60), diameter size by laser diffraction, and determination of Zeta Potential (NanoBrook ZetaPlus Zeta Potential Analyzer from Brookhaven Instruments). It should be noted that all methodologies for characterizing the nanoformulation followed the protocols standardized by Queiroz et al. (2018).

### 2.3. Evaluation of Encapsulation Efficiency (%)

A trypsin inhibition assay was performed using 1.25 mM BApNa (Nbenzoyl-DL-arginine-p-nitroanilide) as a substrate to monitor the incorporation of TTI into the particles [17].

TTI, ECW, and encapsulating agents were used in this assay, considered as controls, to prove the activity from only the encapsulated TTI. The mass of ECW and blanks used was established considering the activity of TTI capable of inhibiting 100% trypsin (1.4 mg). Thus, considering the ratio of TTI to the encapsulating agent (1:2:2 *w*/*w*/*w*), the amount in mg of TTI incorporated was determined based on the percentages of antitrypsin activity.

The encapsulation efficiency was evaluated from the following Formula [19]

Encapsulation efficiency (%) = (TTI into particles/total TTI used) × 100(1)

### 2.4. Effect of Different pH and Temperature Conditions

The assay was conducted to evaluate the potential for chemical interaction of encapsulating agents with TTI in the nanoformulation when exposed to different pHs and temperatures monitored by antitrypsin activity. The activity was assessed in triplicate, using 1.25 mM BApNa (Nbenzoyl-DL-arginine-p-nitroanilide) as substrate [17].

The concentrations used in the assays were established considering the average inhibitory concentration (IC50) of unencapsulated TTI and TTI extracted from ECW to achieve the same percentage of trypsin inhibition, as described by Matias et al. [15]. As controls, non-encapsulated TTI (1.4 mg/mL), ECW (7.0 mg/mL), and TTI extracted from ECW (7.0 mg/mL), which was previously subjected to constant agitation at 300 rpm/12 h at 20 °C (active extraction conditions) (Hettich^®^ MIKRO 200/200R centrifuge), without the proposed exposition, were tested for antitrypsin activity. Therefore, for the exposure tests, TTI (1.4 mg/mL) and ECW (7.0 mg/mL) were used, keeping the same concentration of TTI (1.4 mg/mL) for both, without any previous treatment, exposed to the conditions described below.

#### 2.4.1. pH

The pHs and exposure times were defined, simulating the same phases of digestion in the gastrointestinal tract in humans, as recommended by INFOGEST [20]. Therefore, TTI and ECW were resuspended in 50 mM Tris-HCl buffer, pH 7, and evaluated under the following conditions: oral pH (7.0) for 2 min, gastric pH (3.0) for 2 h, and intestinal pH (7.0) for 2 h. For all these conditions, TTI and ECW were incubated at 37 °C. The pH of 3.0 was adjusted using HCl (1M).

After the exposure time, the antitrypsin activity assay was performed for monitoring [17]. It should be noted that, after gastric exposure, the pH of 7.0 was adjusted using NaOH (6M). For the antitrypsin activity assay, the TTI, ECW, and TTI extracted from ECW, all without passing through the exposures, were considered controls.

#### 2.4.2. Temperature

Temperatures and times were defined as simulating digestion conditions in the gastrointestinal tract of humans, as recommended by INFOGEST [20]. Therefore, TTI and ECW were resuspended in 50 mM Tris-HCl buffer, pH 7, and evaluated under the following conditions: kept in a water bath at 37 °C for 2 and 4 h (Tecnal—Te 056).

In addition, conditions simulating refrigeration temperature (7 °C) for 12 h, three and seven days, and freezing (−18 °C) for 12 h, 15 and 30 days were also evaluated. This exposure time interval was defined from 12 h to the maximum refrigeration and freezing time recommended by CVS ordinance 5, 9 April 2013, Chapter III [21].

After the exposure time, they were returned to room temperature, and the antitrypsin activity assay was performed [17]. For this test, TTI, ECW, and TTI extracted from ECW were not subjected to exposure conditions, being considered as controls.

### 2.5. Experimental Model In Vivo

Adult male rats, weighing 320–420 g, of the Wistar strain, with obesity induced by a high glycemic index diet and high glycemic load (HGLI diet) for 17 weeks, were provided by the vivarium of Universidade Potiguar (UNP, Natal /RN). The animals were kept under standard lighting conditions (12 h light/12 h dark), in cages cleaned weekly, and maintained at a controlled temperature (23–25 °C), with water and food ad libitum.

The experiments were carried out at the Experimental Nutrition Laboratory of the Universidade Potiguar (UNP/RN). The project was approved by the Ethics Committee in the Use of Animals (CEUA-UNP, Natal/RN) under protocol No. 019/2017. The entire experiment was reproduced following the ARRIVE guidelines (Animal Research: Reporting of in Vivo Experiments) [22].

#### 2.5.1. Diet and Treatments

The obese animals were distributed individually and randomly into five groups, being kept for five days in adaptation to establish the conditions of the experiment and the pattern of food consumption, followed by ten days of treatment with diets and interventions.

The standard Labina^®^ diet (produced by Purina do Brasil, considered nutritionally adequate) was purchased commercially. This diet was used as conventional treatment for one of the obese groups and for the ECW-treated group.

The HGLI diet, with a high glycemic index (77.6) and glycemic load (38.8), consisting of the standard Labina^®^ diet, condensed milk, and refined sugar (4.5:4.5:1 *w*/*w*/*w*) [23] was used for the untreated group as well as for the obese groups receiving ECW and TTI.

The concentrations used for TTI (25 mg/kg of weight) and ECW (12.5 mg/kg of weight) were based on previous studies [6,14,15,16]. In this study, the concentration of TTI administered to the animals (2.5 mg/kg) contained in ECW (12.5 mg/kg) was ten times lower compared to the concentration of TTI administered (25 mg/kg) to the animals.

Based on this, the five groups were divided as follows:

1. Untreated obesity (*n* = 5): HGLI diet + 1 mL of water by gavage. The group that did not receive treatment was considered.
2. Obesity treatment 1 (*n* = 5): nutritionally adequate diet (NA diet) (Labina^®^ feed) + 1 mL of water per gavage. The group that received conventional treatment was considered.
3. Obesity treatment 2 (*n* = 5): nutritionally adequate diet (NA diet) (Labina^®^ feed) + 1 mL of ECW (12.5 mg/kg) by gavage.
4. Obesity treatment 3 (*n* = 5): HGLI diet + 1 mL ECW (12.5 mg/kg) by gavage.
5. Obesity treatment 4 (*n* = 5): HGLI diet + 1 mL of TTI (25 mg/kg) by gavage.

#### 2.5.2. Satiety and Zoometric Parameters

During the experiment period (eleven days), the animals received previously weighed portions of their respective diets. Initial consumption (first day), after adaptation and establishment of the individual consumption pattern, and final consumption (eleventh day) were considered. Thus, the variation in dietary intake (Δ) was determined (Equation (2). Residual feed in the cages was not considered, based on Rodrigues et al. [24] with modifications. More precisely, the values were calculated for each animal in the present investigation, and the mean (SD) per group was determined.

Δ Dietary consumption = final consumed weight − initial consumed weight(2)

Regarding weight, the animals were evaluated individually on the same days of the evaluation of food consumption, that is, at the beginning (first day) and the end (eleventh day) of the study, with the mean and standard deviation (SD) being obtained by the group. Thus, determining the variation in body weight (Δ) (Equation (3)).

Δ Weight = final weight − initial weight(3)

Caloric efficiency was determined according to Equations (4) and (5), being 4.184, equivalent to caloric conversion in kilojoules (KJ).

Caloric Intake (KJ) = Final Dietary Consumption (g) × 4.184(4)

Caloric Efficiency = Caloric Intake (KJ) ÷ Body Weight Variation(5)

Weight gain and weight loss (g) were obtained based on Equation (6). It should be noted that this calculation was performed for each rat, obtaining the mean (SD) per group at the end.

Weight loss/gain (g) = final weight − initial weight(6)

A zoometric evaluation comprised the abdominal perimeter, tail length, naso-anal length, and Lee index (cubic root of body weight (g)/naso-anal length × 1000) [25]. The variation in this parameter was obtained based on Equation (7).

Δ Lee Index = Final Lee Index − Initial Lee Index(7)

#### 2.5.3. Evaluation of Biochemical Parameters

After the ten days of the experiment, the animals were submitted to 8 h–12 h of fasting. Subsequently, they were anesthetized (Zoletil^®^) to collect blood for biochemical analysis. Blood collection was performed by cardiac puncture. It should be noted that trained veterinarians performed the application of anesthetic and blood collection.

The blood was centrifuged (500× *g*/10 min at 4 °C) to remove the serum and subjected to fasting glucose, insulin, and triglycerides (TG) analyses. The method used to evaluate the biochemical parameters was the automated enzymatic colorimetric method (Labtest^®^, Natal, RN, Brazil). After collection, the animals were euthanized.

#### 2.5.4. Assessment of Insulin Resistance and Pancreas Activity

After the ten days of the experiment, the animals were subjected to fasting for 8 h–12 h, after which they were anesthetized (Zoletil^®^) to collect blood for biochemical analyses. Blood collection was performed by cardiac puncture. It should be noted that trained veterinarians performed the application of anesthetic and blood collection.

The homeostatic model assessment for insulin resistance (HOMA-IR) was calculated using the following formula (Equation (8) [26]

HOMA-IR: fasting insulin (μIU/mL) × fasting glucose (mmol/L)/22.5(8)

Insulin sensitivity was assessed using the quantitative verification of the insulin sensitivity index (QUICKI) using the following formula (Equation (9)) [26]

QUICKI = 1/[log(I₀) + log(G₀)](9)

I₀ = fasting insulin expressed in µU/mL.

G₀ = fasting glucose expressed in mg/dL.

The homeostatic model assessment of the index of beta cell function (HOMA-BETA) was calculated using the following formula (Equation (10)) [26]

HOMA β = (20 × Insulin μIU/mL)/(Glucose mmol/L − 3.5)(10)

#### 2.5.5. Inflammatory Markers and Leptin

For the dosage of TNF-α, IL-6, and leptin, the serums were analyzed using commercially available immunoassay kits, as proposed by Carvalho et al. [6]. TNF-α was determined using the Quantikine Mouse TNF-α immunoassay kit (R&D Systems # RTA00), and IL-6 was measured using the Quantikine Mouse IL-6 Immune Kit (R&D Systems # R6000B).

According to the manufacturer, the sensitivity of the kits was <15 pg/mL and <30 pg/mL for TNF-α and IL-6, respectively. Leptin concentration was determined in the plasma of animals after euthanasia. Plasma was separated by centrifugation at 3000 rpm for 15 min at 4 °C. Leptin was quantified using the mouse leptin ELISA kit (millipore^®^-EZRL83K).

### 2.6. Statistical Analysis

The sample size was calculated considering a simple and random sampling (Cochran model [27]) and according to the 3Rs principle (replacement, reduction, and refinement). The result was an “*n*” equal to 4.36 (5) animals per group. For this, a physiologically significant difference in the parameters evaluated was assumed when the treatment (ECW) exerted a biological effect of 25% or more concerning the group that did not receive the diet. Furthermore, an anticipated coefficient of variation of 10% was adopted, with an error probability lower than 5% and a power of 90%.

The results obtained in different temperature, pH, and time conditions were evaluated using the Student’s *t*-test to compare TTI and ECW. Data from the in vivo experiment were analyzed for normality using the Kolmogorov–Smirnov test. For the parametric variables, ANOVA and Tukey’s post-test were used. In contrast, for the non-parametric variables, the Kruskal–Wallis test and Dunns’ post-test were applied to identify whether the biochemical and inflammatory parameters data differed between the groups tested.

Data were analyzed using GraphPad Prism, version 5.0 (Graph Pad Software, San Diego, CA, USA). For all tests performed, values of *p* < 0.05 were considered significant.

## 3. Results

### 3.1. Isolation of the Trypsin Inhibitor from Tamarind Seeds (TTI)

**Figure 1:**
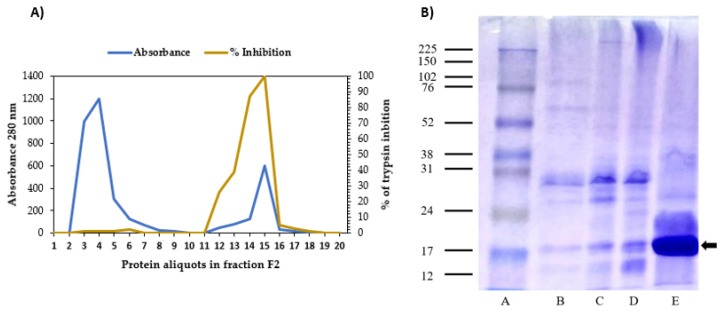
(**A**) Isolation of trypsin inhibitor from tamarind seeds. A. Chromatographic profile and antitrypsin activity of protein fraction 2 of tamarind seeds by CNBr 4B Trypsin-Sepharose Affinity Chromatography. The blue line represents aliquots of proteins monitored at 280 nm by spectrophotometry, the first peak being the elution of unretained material, and the second peak, the proteins adsorbed on the matrix eluted with HCl (5 mM). The brown line represents the antitrypsin activity, using 100 µL of TTI and 1.25 mM BApNA (Nbenzoyl-DL-arginine-p-nitroanilide) as substrate. (**B**) This shows 12.5% denaturing polyacrylamide gel electrophoresis (SDS-PAGE) stained with Coomassie blue R-250. A. Molecular Weight Marker Amersham™ ECL™ Rainbow™ Marker—Full range GE Healthcare; B. Crude Extract; C. Protein fraction 1 (saturation using 0–30% ammonium sulfate); D. Protein fraction 2 (saturation with 30–60% ammonium sulfate); E. TTI (tamarind trypsin inhibitor) (arrow).

The SDS-PAGE gel showed that TTI was isolated, which can be confirmed by protein bands with a predominance of molecular mass around 20 kDa (Figure 1a). Furthermore, it was observed that 1.4 mg of TTI could promote 100% (1810 IU/mg) inhibition against trypsin by determining antitrypsin activity.

### 3.2. Synthesis and Physicochemical Characterization of the ECW

**Figure 2:**
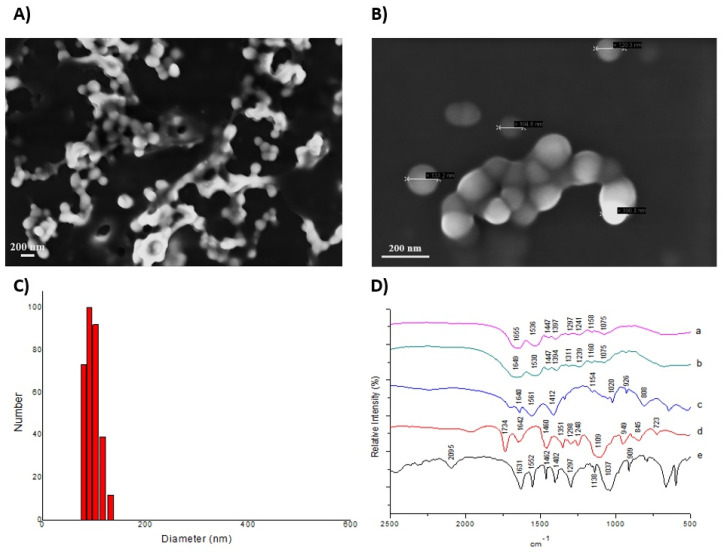
Characterization of ECW. (**A**) Scanning Electron Microscopy (SEM) with a magnitude of 20.00 KX, a working distance of 4.0 mm, and an acceleration voltage of 3.00 kV. (**B**) Scanning Electron Microscopy (SEM) with a magnitude of 80.00 KX, a working distance of 4.0 mm, and an acceleration voltage of 3.00 kV. (**C**) Laser diffraction obtained by crosslinking using formaldehyde and dispersing ECW in water. (**D**) Fourier Transform Infrared Spectroscopy (FTIR). ECW: TTI encapsulated in purified chitosan and whey protein isolate (1:2:2 *w*/*w*/*w*). (a) EWPI: Whey protein isolate; (b) ECH: purified chitosan; (c) Tween 80; (d) TTI: tamarind trypsin inhibitor; (e) ECW: TTI encapsulated in purified chitosan and whey protein isolate (1:2:2 *w*/*w*/*w*).

The micrographs (Figure 2a,b) show that the particles appear agglomerated with a spherical shape, smooth surface, and physical size on a nanometric scale.

According to laser diffraction, the mean (SD) diameter of ECW dispersed in water (Figure 2c) was 109 nm (6.7), and the polydispersity index was 0.406 (0.05). The Zeta Potential revealed an average surface charge of −38.42 (0.25). For incorporation efficiency, the results showed a high incorporation capacity of TTI in the nanoparticles, with an efficiency of 95.19% (0.38).

The FTIR spectra show the chemical interactions of TTI with purified chitosan and isolated whey protein (Figure 2d), verified by the appearance of a new vibrational band (1241 cm^−1^) from the attenuation of the bands referring to the presence of TTI (1297 cm^−1^) and characteristic bands of whey protein isolate (1075 and 1447 cm^−1^), as well as the displacement of the characteristic bands of this protein (1158 cm^−1^).

### 3.3. ECW Assessment under Different pH and Temperature Conditions

Antitrypsin activity assays were performed to determine the effect of pH, and physiological and storage temperatures on ECW in the interaction of its encapsulating agents with TTI. All tests were performed using the same concentrations of TTI contained in or extracted from ECW. For the antitrypsin activity assay, TTI, ECW, and TTI extracted from ECW, without passing through the exposures, were considered controls.

### 3.4. Influence of pH

After the exposure time, aliquots of TTI and ECW exposed to oral, gastric, and intestinal pH were collected and evaluated for their ability to inhibit trypsin (%) (Table 1).

TTI showed high antitrypsin activity at oral pH (100.00% (1.27)), gastric pH (100.00% (0.83)), and intestinal pH ((100.00% (1. 07)). Considering the antitrypsin activity of the TTI ((100.00% (0.66)) used as a control (without exposition), it was noticed that the TTI did not suffer significant action in the pH ranges evaluated compared to the control group.

This same test performed with the ECW showed different percentages of antitrypsin activity, 0.0% (0.0), 44.07% (1.03), and 20.10% (2.85), referring to exposure to oral, gastric, and intestinal pHs, respectively. The antitrypsin activity of the TTI extracted from the ECW was reported as a control, showing a high antitrypsin activity, 100.00% (0.48) (Table 2). As control groups for antitrypsin activity, ECW without the extraction procedure of TTI was 0.0% (0.00) and 100% (0.48) when TTI was previously extracted from ECW.

In this case, it was evident that the absence of activity against trypsin or even the low percentage of ECW inhibition indicated that it protected the TTI in the investigated condition. Evidently, in the exposure of ECW to gastric pH, it can be noticed that there was some release of TTI.

### 3.5. Effect of Physiological Temperature

The temperature of 37 °C did not affect the antitrypsin activity of TTI at 2 h and 4 h of exposure (*p* > 0.05). For ECW, during 2 h at 37 °C, an antitrypsin activity of 38.62% (0.14) was observed (Table 2). By increasing the exposure time from 2 h to 4 h, this activity was higher (94.42% (1.52)), demonstrating that the TTI was possibly being released as time passed (*p* < 0.05). These data showed that the temperature of 37 °C influenced the TTI release, and the release became greater with the time of this exposure.

### 3.6. Storage Temperature Effect

In the studied exposure times, the temperature conditions of 7 °C and −18 °C did not affect the antitrypsin activity of TTI (*p* > 0.05). However, it directly impacted the ECW since 12 h of exposure at 7 °C was sufficient to detect 68.70% (4.38) antitrypsin activity (Table 2). Furthermore, the exposure time significantly increased the presence of antitrypsin activity, and in 7 days, this detection was 100% (0.97). The temperature change maintained the sustained release of TTI (*p* > 0.05). TTI as a control of activity without the exposure showed 100% (0.66)

In 12 h at −18°C, ECW antitrypsin activity was 52.90% (3.67) (Table 2). By increasing the exposure time to 30 days, this activity increased to 83.56% (2.9), demonstrating that the TTI was being released as time passed (*p* < 0.05). As controls for antitrypsin activity, ECW without the extraction procedure of TTI was 0.0% (0.00) and 100% (0.48) when TTI was extracted from ECW. These data showed that the freezing temperature influenced the release of TTI and, consequently, the increase in antitrypsin activity, depending on the exposure time. The higher the temperature, the more it controlled the release.

### 3.7. Variation in Dietary Intake, Caloric Intake, Caloric Efficiency, Body Weight, and Nutritional Status

**Figure 3:**
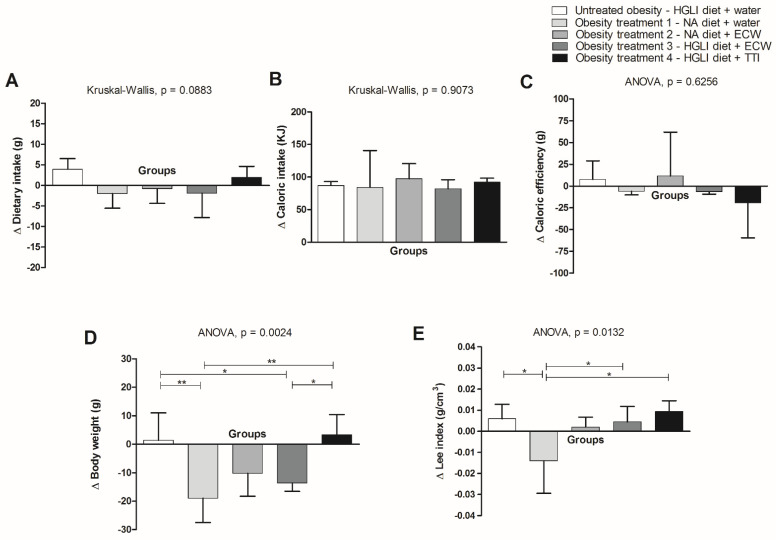
Variation in dietary intake, caloric intake, caloric efficiency, body weight, and nutritional status of Wistar rats with obesity induced by HGLI diet, submitted for ten days to different treatments. (**A**). Dietary intake variation = final consumed weight—initial consumed weight. (**B**). Caloric intake (KJ) = final dietary intake (g) × 4.184. (**C**). Caloric Efficiency = Caloric Intake (KJ) ÷ Body Weight variation. (**D**). Weight Loss/Gain Variation (g) = final weight—initial weight. (**E**). Lee index = cubic root of body weight (g)/naso-anal length × 1000). HGLI diet: high glycemic index diet and high glycemic load, a mixture composed of Labina^®^, condensed milk, and sugar (1:1:0.21 *w*/*w*/*w*); NA diet: nutritionally adequate diet (standard diet) (Labina^®^); TTI: tamarind trypsin inhibitor; ECW: TTI encapsulated in purified chitosan and whey protein isolated (1:2:2 *w*/*w*/*w*). Values are expressed as mean (standard deviation). Equal letters indicate no significant difference between the groups evaluated for each parameter. The Kolmogorov–Smirnov test was used to assess the normality of the data, the statistical difference using the Kruskal–Wallis test, and the Dunn’s post-test (*p* < 0.05) for variation in consumption (∆) and variation in caloric intake (∆), and by ANOVA and Tukey’s post-test (*p* < 0.05) for the changes in caloric efficiency (∆), weight variation (∆), and Lee index variation (∆). Values of *p* ≤ 0.05 were considered statistically significant (* *p* < 0.05; ** *p* < 0.01).

The analysis of body weight variation indicated a greater weight reduction in animals treated with a nutritionally adequate diet, followed by animals treated with ECW, having been administered with the HGLI or nutritionally adequate diet, than untreated animals (HGLI diet and water gavage) (Figure 3d). Thus, it was demonstrated that the encapsulation potentiated the action of TTI on this parameter (body weight).

This more significant weight loss is probably due to the negative mean variation in dietary intake (Figure 3a) and, consequently, to the lower mean variation in intake and efficiency (Figure 3b,c), even though the evaluated groups did not show a significant difference for these parameters (*p* > 0.05). Moreover, this negative weight variation was insufficient to reduce the Lee index (Figure 3e) (*p* > 0.05). However, the variation in the average of the Lee index of the animals treated with ECW was smaller than that presented by the animals treated with TTI (Figure 3e).

Similarly, treatments with the nutritionally adequate diet alone or associated with ECW or the nanoformulation associated with the HGLI diet were the three treatments that only promoted weight loss. This was different from the treatment with TTI, which led to weight gain in the animals, even though this gain was lower than that achieved by untreated animals (Table 3).

### 3.8. Evaluation of Biochemical Parameters

**Figure 4:**
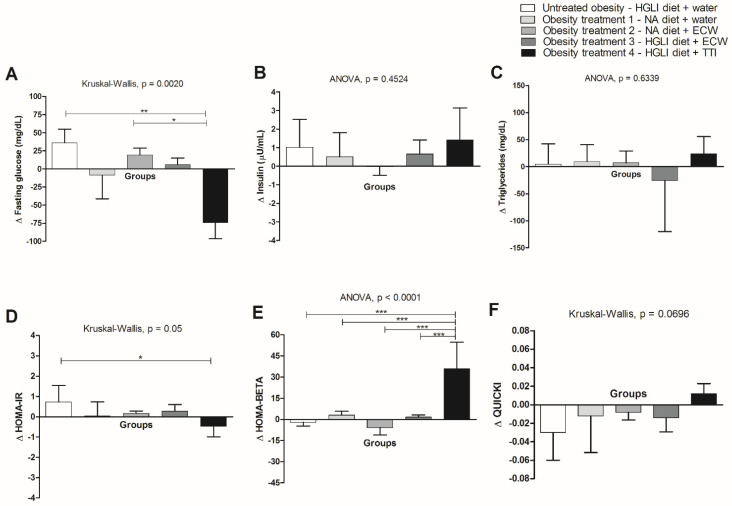
Biochemical parameters in obese Wistar rats submitted for ten days to different types of treatment: (**A**). Fasting glucose. (**B**). Insulin. (**C**). Triglycerides. (**D**). HOMA-IR. (**E**). HOMA-BETA. (**F**). QUICKI. HGLI diet: high glycemic index diet and high glycemic load high glycemic index and high glycemic load diet, a mixture composed of Labina^®^, condensed milk, and sugar (1:1:0.21 *w*/*w*/*w*); NA diet: nutritionally adequate diet (standard diet) (Labina^®^); TTI: tamarind trypsin inhibitor; ECW: TTI encapsulated in purified chitosan and whey protein isolate (1:2:2 *w*/*w*/*w*). Values are expressed as a mean (standard deviation). The Kolmogorov–Smirnov test was used to assess data normality. The statistical difference was evaluated using the Kruskal–Wallis test and the Dunn’s post-test (*p* < 0.05) for blood glucose variations (∆), HOMA-IR (∆) and QUICKI (∆), and by ANOVA and Tukey’s post-test (*p* < 0.05) for variations in insulin (∆), triglycerides (∆) and HOMA-BETA (∆). Values of *p* ≤ 0.05 were considered statistically significant (* *p* < 0.05; ** *p* < 0.01; *** *p* < 0.0001).

For most of the biochemical parameters evaluated, there were no significant differences when comparing the groups of animals treated with ECW and TTI (Figure 4a). However, for fasting glucose (Figure 4a) and HOMA-BETA (Figure 4f), there was a significant difference between animals treated with TTI and the HGLI diet and ECW and the nutritionally adequate diet (Figure 4a). The animals treated with ECW, even with lower means for fasting glucose compared to untreated animals, were not significantly different from these animals (*p* > 0.05). However, the untreated animals were different from the animals treated with TTI (*p* < 0.05), which showed a negative mean variation for fasting glucose (Figure 4a), independent of insulin (Figure 4b), because there were no differences between treatments.

Therefore, only the treatment with TTI showed a significant difference in HOMA-IR compared to the untreated group, the only one with a negative variation for this parameter (Figure 4d). These results impacted the significant difference found for animals treated with TTI and all other groups in the HOMA-BETA analysis. For QUICKI, all groups showed a negative variation (Figure 4f), except for animals treated with TTI but without significant differences. Another highlight can be given to the group treated with ECW and the HGLI diet, which showed a negative mean variation for triglycerides but without significant differences between the other groups (Figure 4c).

### 3.9. Evaluation of Biochemical Parameters

**Figure 5:**
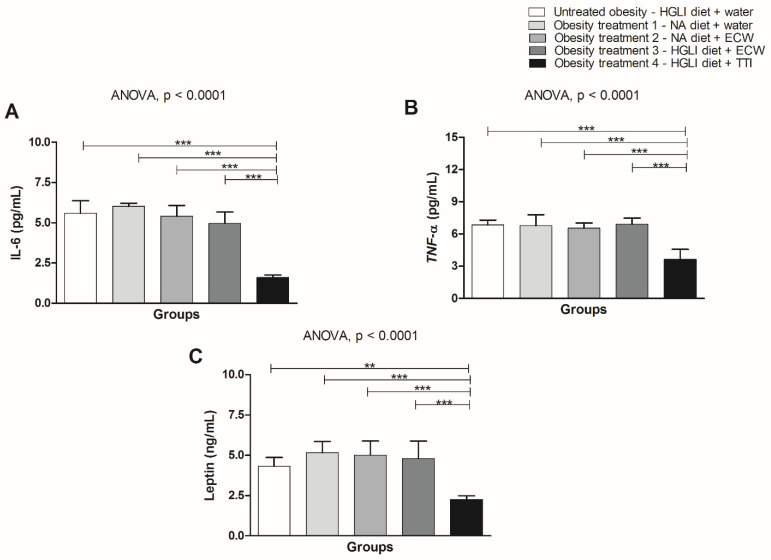
Inflammatory parameters and leptin dosage in obese Wistar rats submitted for ten days to different types of treatment: (**A**). IL-6; (**B**). TNF-α; (**C**). Leptin. HGLI diet: high glycemic index diet and high glycemic load high glycemic index and high glycemic load diet, a mixture composed of Labina^®^, condensed milk, and sugar (1:1:0.21 *w*/*w*/*w*); NA diet: nutritionally adequate diet (standard diet) (Labina^®^); TTI: tamarind trypsin inhibitor; ECW: TTI encapsulated in purified chitosan and whey protein isolate (1:2:2 *w*/*w*/*w*). Values are expressed as mean (standard deviation). The Kolmogorov–Smirnov test was used to assess data normality. The statistical difference was evaluated using ANOVA and Tukey’s post-test (*p* < 0.05) for IL-6, TNF-α, and Leptin. Values of *p* ≤ 0.05 were considered statistically significant(; ** *p* < 0.01; *** *p* < 0.0001).

The results referring to the dosage of inflammatory markers (IL-6 and TNF-α), and leptin, show that only the group treated with TTI showed a significant difference (*p* < 0.05) with the lowest plasma concentrations of these cytokines compared to the other groups (Figure 5a–c).

## 4. Discussion

Given the potentialities observed and attributed to TTI, using nanoencapsulation has become an attractive strategy to validate the use of this molecule for complementary studies in in vivo and in vitro models since potentiating the bioactive activities of TTI may enable new applications for managing obesity or its complications.

Queiroz et al. [13] synthesized and characterized ECW, and, posteriorly, other preclinical studies were conducted with this nanoformulation using the same method of obtaining [14,15,16]. In the present study, TTI and ECW presented the same physicochemical characteristics and encapsulation efficiency, similar to previous studies [5,6,7,9,13,14,15,16]. Thus, it demonstrated the method’s reproducibility and guaranteed it was the same nanoparticle in question.

Studies of the NutriSBioativos research group had not yet investigated the effects on the stability of the antitrypsin activity of ECW compared to TTI against different physiological pHs (without the influence of enzymatic action) and physiological and storage temperatures, considering exposure at different times. Thus, there is a need for a better understanding of the applicability perspective of nanoparticles containing TTI, making it possible to suggest its future use in food and pharmaceutical products.

At different temperature, pH, and time conditions, it was observed that ECW, without any exposure, did not show antitrypsin activity, unlike TTI, which showed high activity against trypsin (100%). These data confirm that ECW nanoparticles encapsulated and protected TTI without showing residual inhibitory activity (0.0%). However, when ECW was subjected to active extraction conditions (constant agitation at 300 rpm for 12 h), inhibitory activity against trypsin was observed [(100.00% (0.48)].

Regarding this technique, it is noticeable that the particles kept the TTI protected, and when submitted to mechanical stress, the inhibitor was exposed to interaction with trypsin chemically. Inhibitor confinement comes from solid chemical interactions between chitosan and TTI, and these interactions are highlighted in previous studies [13,14,15]. Hydrogen bonds and Van Der Walls forces are examples of interactions in ECW. Based on this information, it is noticeable that the interactions did not prevent the release of the active (TTI), nor did they influence the antitrypsin activity of the TTI, which is essential since the bioactive properties of the inhibitor are due to its activity in the free form.

With the proposal to enable the application of particles containing the inhibitor, tests are necessary to detect factors limiting the application in food and pharmaceutical products [12]. Thus, this study verified, under various physiological and environmental conditions, such as exposure to short, medium, and long-term temperatures and exposure to different pHs, the effect in TTI present in ECW, which can influence its use in foods or even as a vehicle for the pharmaceutical industry.

Thus, ECW was exposed to different pHs, simulating the conditions of oral (pH 7), gastric (pH 3), and intestinal (pH 7) digestion. ECW, in this study, proved to be an excellent vehicle for TTI for oral administration, as it remained protected and was released only under conditions that simulated digestion’s gastric and intestinal phases.

The fact that the presence of the inhibitor has activity at acidic pH is crucial since all the works with animals already carried out with these molecules, TTI or ECW, were administered by gavage, where the first contact was the stomach. Therefore, the presence of TTI, released from ECW, in this pH range would guarantee its proteolytic hydrolysis and, consequently, the absorption of its derived peptides and the systemic effects already attributed to it, whether on satiety, anti-inflammatory activity, or as a hypoglycemic agent [6,7,9].

Regarding the results obtained for ECW, previous studies have already found its antitrypsin activity in exposure to acidic pH and, therefore, a destabilization of the interactions allowing the detection of the released TTI. However, the stability of the electrostatic interactions present in the system during exposure to neutral pH was observed, as the TTI remained confined [14]. Although the particles were not dispersed in the present study at the mentioned pH, the TTI was kept confined in Tris-HCl buffer (pH 7.5), as observed by Costa et al. [14].

Because of the acidic pH after exposure for 2 h, there was also some release of TTI. Matias et al. [15] observed this same event simulating in vitro digestion by the ECW gastrointestinal tract, where antitrypsin activity was predominant in the gastric phase, confirming the release of TTI.

Not only does the pH influence changes in the interactions between the active and its encapsulating agents, impacting the formation of pores in the particle, but it can also be altered under the influence of other environmental conditions, such as temperature [28]. The ECW had already been evaluated for temperature [13]. However, the potential for system interactions at cold storage temperatures and longer exposure times was not assessed.

The present study evaluated the effects of physiological temperature (37 °C), refrigeration (7 °C), and freezing (−18 °C) on ECW antitrypsin activity. During the exposure phase at 37 °C, detection of antitrypsin activity was observed at 2 h, which was time-dependent, as it was more pronounced at 4 h. However, TTI remained with high antitrypsin activity, showing that the inhibitor was still intact after release. These data show instability in the system, possibly affecting the chemical interactions between chitosan and whey protein, with the consequent release of TTI. It is possible to verify that the TTI, when released from ECW, maintains the thermoresistance at the temperatures evaluated, already observed for the TTI [8]. In the study by Queiroz et al. [13], the temperature of 40 °C led to the release of TTI, and it remained with antitrypsin activity up to 80 °C.

During exposure to heating, proteins are susceptible and change their structure. Native globular proteins unfold, exposing hydrophobic regions and causing solid electrostatic repulsion [29]. Since whey proteins are mostly globular and therefore sensitive to thermal processing, it is assumed that in this study, this caused the inhibitor to be more exposed to interact with the medium and act by inhibiting the trypsin.

For ECW, under the same conditions of temperature and storage time under refrigeration or freezing, TTI was released, also showing high percentages of inhibition against trypsin, similar to those presented by TTI. This release was time-dependent since as this time was prolonged, antitrypsin activity increased, which suggests that refrigeration and freezing conditions as well, as already reported for exposure to 37 °C, can impact electrostatic interactions of the ECW.

The release of proteins is a point of interest for the pharmaceutical industry since this effect, in addition to promoting greater stability, is associated with improving the encapsulated active’s bioactive function [30]. Thus, these findings reveal promising evidence for the use of nanoparticles containing TTI, whether for the food industry or as a vehicle in the pharmaceutical industry. 

Considering the protection of TTI observed in ECW in the present study and others, in addition to the low toxicity [13,14,15] and the bioactive potential of ECW evidenced in animals with obesity [15,16], in the present study, we investigated and compared the effects of TTI and ECW on dietary intake, weight, Lee index, biochemical, and inflammatory parameters in Wistar rats with diet-induced obesity.

In the literature, TTI has been shown to reduce food consumption and weight in eutrophic and obese Wistar rats [5,6,7]. However, for obese animals, TTI reduced appetite without significantly influencing weight [6].

Protein encapsulation has been an excellent strategy to potentiate the effects, in addition to those already reported for TTI [13,15,16]. This study was the first to compare the impact of TTI and ECW. It showed significant weight loss (*p* < 0.05) in the group treated with the nutritionally adequate diet and treated with ECW, administered together with both tested diets. Thus, this significant difference is believed to point to a new action of the inhibitor. This functionality was not observed in the treatment with TTI in obese animals, and the animals gained weight, as already observed in previous studies with TTI.

Trypsin inhibitors can prolong the action of some hormones, acting as secretagogues, including cholecystokinin (CCK) [31]. Several factors, including CCK, impact the signaling of satiety at the intestinal level. The CCK action depends on the presence of nutrients in the diet and the stimulation of protein factors in the intestinal lumen. The impact on the hormone action is influenced by the fed state (post-prandial) and the trypsin inhibitor’s presence, contributing to the prolongation of its activity and, consequently, its effect on satiety [2,5,31,32].

It is known that a sustained release of proteins could modulate this action on satiety by prolonging the action of CCK [33]. Studies show that the encapsulation of peptides and proteins impacts the body weight of rats by modulating hormones without directly impacting food consumption [34]. Thus, it is assumed that ECW, releasing TTI sustainably, would prolong the action of CCK, directly impacting food consumption and consequent weight loss. However, this effect has already been observed in previous studies using TTI in eutrophic animals [5] but not in obese animals [6,7]. However, in this study, it was not possible to assess the action of ECW on CCK, and thee data are necessary to confirm this hypothesis in future studies.

Regarding the biochemical parameters evaluated, it was noticed that there were no significant differences in the plasma concentrations of insulin and triglycerides between the animals treated with TTI and ECW (*p* > 0.05). Only the animals treated with TTI, concerning fasting glucose, differed significantly from the group of untreated animals. Moreover, for fasting glucose, the group of animals treated with TTI was significantly different (*p* < 0.05) from those treated with ECW and a nutritionally adequate diet.

It is understood that, in this case, the nanoencapsulation did not potentiate the action of TTI significantly. However, ECW contained ten times less TTI; therefore, comparing the dose administered to the animals, this was much higher than the contained dose in ECW. Matias et al. [15] and Aguiar et al. [16] had already documented the hypoglycemic effect of ECW and that it was insulin-independent, without comparing it to TTI however. Costa et al. [14] found a lower fasting glucose concentration for animals treated with TTI. In this study, comparing the effect of TTI and ECW, it is believed that the hypoglycemic effect of ECW, documented in previous studies, occurred through TTI when released and that in this circumstance, it was more potent to promote this effect, as it needed ten times less TTI.

Compared to ECW, this activity was not observed in this study related to the anti-inflammatory aspect of TTI. The combination of encapsulating agents in the system, such as chitosan, may have influenced the three-dimensional rearrangement of the inhibitor or influenced the generation of peptides responsible for this activity.

This corroborates the report of the study by Tilkin et al. [35], which encapsulated a soybean trypsin inhibitor with silica by the sol–gel method, which led to changes in protein conformation with preservation of activity. According to the authors, variations were observed depending on the conditions tested during time exposures evaluating the release kinetics and trypsin inhibitory activity. By subjecting the encapsulated to a prolonged exposure time, part of the inhibitory activity was lost. The authors explain that changes in the protein structure could preserve or lead to the loss of this activity. Thus, it is believed that this would be the reason for the absence of the anti-inflammatory effect of the TTI when not encapsulated (ECW) but that it did not negatively affect the antitrypsin activity, which is essential for the satietogenic effect [5]. Moreover, the impact of ECW on body weight reduction was found in this study on obese animals. In addition, the dose of TTI present in ECW could also affect the anti-inflammatory action, as highlighted above.

## 5. Conclusions

The data obtained in the present study showed the potential effect that ECW had on TTI due to nanoencapsulation, allowing a smaller amount of the inhibitor to promote biological effects, which was more potent on weight loss. In addition to studies to evaluate the impact of different temperature and time conditions on antitrypsin activity, even in the face of solid interactions of encapsulating agents with the inhibitor, ECW allows the release of TTI, which did not affect antitrypsin activity. Furthermore, this antitrypsin activity seems to be related to lower weight gain in obese animals treated with ECW, considering the relationship of this activity with CCK and, consequently, its effect on satiety. Thus, ECW is promising for future clinical applications to treat or manage obesity and its complications.

## Figures and Tables

**Table 1:** Comparison of the percentage of antitrypsin activity between ECW and TTI exposed to different conditions of pH and time.

Samples	Exposed to Oral pH (7.0) Mean (SD) (%)	Exposed to Gastric pH (3.0) Mean (SD) (%)	Exposed to Intestinal pH (7.0) Mean (SD) (%)
TTI	100 (1.27) ^A,a^	100 (0.83) ^A,a^	100 (1.07) ^A,a^
ECW	0.00 (0.00) ^B,a^	44.07 (1.03) ^B,b^	20.10 (2.85) ^B,c^

Antitrypsin activity was performed using 100 µL and 1.25 mM BApNA (Nbenzoyl-DL-arginine-p-nitroanilide) as substrate. TTI: tamarind trypsin inhibitor; TTI encapsulated in purified chitosan and whey protein isolate (1:2:2 *w*/*w*/*w*); TTI extracted from ECW: subjected to constant agitation at 300 rpm for approximately 12 h at 20 °C. Mean (standard deviation), *n* = 3. Same capital letters in the same column: means did not differ significantly according to the T-Student test (*p* > 0.05). Equal lowercase letters on the same line: the means did not differ significantly according to ANOVA and Tukey’s post-test (*p* > 0.05).

**Table 2:** Comparison of the percentage of antitrypsin activity between ECW and TTI exposed to different temperature and time conditions.

Samples	Exposed to 37 °C (2 h) Mean (SD) (%)	Exposed to 37 °C (4 h) Mean (SD) (%)	Exposed to 7 °C (12 h) Mean (SD) (%)	Exposed to 7 °C (3 Days) Mean (SD) (%)	Exposed to 7 °C (7 Days) Mean (SD) (%)	Exposed to −18 °C (12 h) Mean (SD) (%)	Exposed to −18 °C (15 Days) Mean (SD) (%)	Exposed to −18 °C (30 Days) Mean (SD) (%)
TTI	92.00 (0.39) ^A,a^	94.00 (1.50) ^A,a^	90.90 (4.47) ^A,a^	95.50 (1.14) ^A,a^	95.70 (4.02) ^A,a^	91.45 (1.18) ^A,a^	95.80 (0.42) ^A,a^	100 (2.22) ^A,a^
ECW	38.62 (0.14) ^B,a^	94.42 (1.52) ^A,b^	68.70 (4.38) ^B,a^	91.20 (8.03) ^A,b^	100 (0.97) ^A,b^	52.90 (3.67) ^B,a^	46.40 (4.10) ^B,a^	83.60 (2.90) ^B,b^

Antitrypsin activity was performed using 100 µL and 1.25 mM BApNA (Nbenzoyl-DL-arginine-p-nitroanilide) as substrate. TTI: tamarind trypsin inhibitor; ECW: TTI encapsulated in purified chitosan and whey protein isolate (1:2:2 *w*/*w*/*w*); TTI extracted from ECW: subjected to constant agitation at 300 rpm for approximately 12 h at 20 °C. Mean (standard deviation), *n* = 3. Same capital letters in the same column: means did not differ significantly according to the T-Student test (*p* > 0.05). Equal lowercase letters on the same line: the means did not differ significantly according to the T-Student test (*p* > 0.05).

**Table 3:** Body weight variation in Wistar rats with obesity induced by HGLI diet submitted for ten days to different types of treatments.

Evaluated Groups	Δ Body Weight Mean (SD)	Weight Loss (g)	Weight Gain (g)
Untreated obesity	1.40 (9.61) ^A^	−8.00	7.70
Obesity treatment 1	−19.00 (8.52) ^B^	−19.00	-
Obesity treatment 2	−10.20 (5.89) ^A,B^	−13.00	-
Obesity treatment 3	−13.60 (2.97) ^B^	−13.60	-
Obesity treatment 4	−3.33 (9.19) ^A^	-	3.00

Untreated obesity: HGLI diet; **Obesity treatment 1:** NA diet (Labina^®^ feed) + 1 mL of water by gavage; **Obesity treatment 2:** NA diet + 1 mL ECW (12.5 mg/kg) by gavage; **Obesity treatment 3:** HGLI diet + 1 mL ECW (12.5 mg/kg) by gavage; **Obesity treatment 4:** HGLI diet + 1 mL of TTI (25 mg/kg) by gavage. HGLI diet: high glycemic index diet and high glycemic load, a mixture of Labina^®^, condensed milk, and sugar (1:1:0.21 *w*/*w*/*w*); NA diet: nutritionally adequate diet (standard diet) (Labina^®^); TTI: tamarind trypsin inhibitor; ECW: TTI encapsulated in purified chitosan and whey protein isolate. Values are expressed as mean (standard deviation). Equal capital letters indicate no significant difference between the groups evaluated for each parameter. The Kolmogorov–Smirnov test was used to assess data normality. Body weight variations (∆) showed a parametric distribution, so ANOVA and Tukey’s post-test were used to verify the difference between the evaluated groups. *p*-values ≤ 0.05 were considered statistically significant.

## Data Availability

The data presented in this study are available on request from the corresponding author.
